# 12-Month prevalence of allergies in Germany

**DOI:** 10.17886/RKI-GBE-2017-015

**Published:** 2017-03-15

**Authors:** Roma Schmitz, Ronny Kuhnert, Michael Thamm

**Affiliations:** Robert Koch Institute, Department for Epidemiology and Health Monitoring, Berlin, Germany

**Keywords:** ALLERGIES, ADULTS, GERMANY, HEALTH MONITORING, GEDA

## Abstract

The prevalence of allergies has increased dramatically during recent decades, and thus, got into the focus of public health. As part of the 2014 German Health Update (GEDA 2014/2015-EHIS), 28.1% of respondents reported that they were affected by an allergic disease other than asthma. Reports of allergies are more common among women than men and among younger and middle-aged adults than people over the age of 65. Adults with higher levels of education stated more frequently that they are affected by allergies than adults with lower levels of education. Allergic reactions occur in various organ systems, but the skin, mucous membranes, respiratory tract and the intestines are most commonly affected. As allergic reactions often significantly restrict people’s quality of life, early diagnosis and appropriate care for sufferers is essential.

## Introduction

Well-known symptoms of allergies include a runny nose, sneezing, itchy eyes, breathing difficulties up to shortness of breath, and severe itching of the skin. Allergic reactions are triggered by an excessive reaction of the body’s immune system to otherwise harmless environmental substances (allergens). Allergens are widespread and people are exposed to them through inhalation, diet and direct contact; they normally consist of proteins or protein compounds but have a variety of chemical and physical characteristics. Allergic reactions occur in a range of the body’s organ systems, with the skin and mucous membranes (hay fever, atopic dermatitis and allergic contact dermatitis), the respiratory tract (asthma), the oral cavity and the intestinal tract (food allergies) the most commonly affected. Type I reactions (immediate hypersensitivity) such as hay fever and atopic dermatitis involve the production of allergen-specific immunoglobulin E antibodies (IgE). Allergic sensitisation (atopy) is said to have occurred if these antibodies are detectable in the blood. In other allergic disorders, such as allergic contact dermatitis, an allergic reaction is delayed (Type IV hypersensitivity), and is mediated by specific white blood cells (T-cells) [1].

Since the 1970s, allergies have become significantly more common in Germany [2-7]. Estimates suggest that up to 30 million people are currently affected, and their quality of life and capabilities can be greatly restricted [8]. Moreover, chronic allergic disorders are usually associated with a high degree of care needs. In cases where it is impossible or very difficult to avoid exposure to specific allergens, allergy sufferers – depending on the extent of their disorder – remain reliant on medical treatment for their individual complaints, or specific immunotherapy as the only existing form of causal therapy so far. No detailed calculations of the costs of allergic conditions in total are available for Germany.

**GEDA 2014/2015-EHIS**

**Table:** 

**Data holder:**	Robert Koch Institute
**Aims:**	to provide reliable information about the population’s health status, health-related behaviour and health care in Germany, with the possibility of a European comparison
**Method:**	questionnaires completed on paper or online
**Population:**	people aged 18 years and above with permanent residency in Germany
**Sampling:**	registry office sample; randomly selected individuals from 301 communities in Germany were invited to participate
**Participants:**	24,016 people (10,872 men; 13,144 women)
**Response rate:**	26.9%
**Study period:**	November 2014 – July 2015
**Data protection:**	all participants were informed about the study’s aims and content and about data protection, and provided their informed consent

More information is available at

www.geda-studie.de

## Indicator

In the 2014 German Health Update (GEDA 2014/2015-EHIS) the prevalence of allergic disease in the 12-month period that preceded the study was defined by a positive answer to the question ‘Have you had allergy, such as rhinitis, hay fever, eye inflammation, dermatitis, food allergy or other (allergic asthma excluded) in the past 12 month?’ This indicator describes 12-month prevalence of self-assessed current affection by an allergic disease other than asthma.

Analysis is based on data from 23,342 participants aged 18 and above with available information about their 12-month allergy prevalence (674 participants were excluded from the analysis because of missing information). A weighting factor was applied throughout statistical analysis that corrected the sample for deviations from the German population (as of 31 December 2014) in terms of gender, age, type of municipality and level of education. The article entitled German Health Update – New data for Germany and Europe [9], published in this issue, provides a detailed description of the study methodology. A detailed description of health monitoring and health indicators in Europe is provided by a Focus article [10] that is also published in this issue.

## Results and discussion

28.1% of the adults reported that they are currently affected by allergies other than allergic asthma. Women – at 31.6% – are significantly more likely to report that they are affected by allergies than men – at 24.5%. Young and middle-aged adults (up to 65 years of age) more frequently report that they are affected by allergies than older people. Adults with higher levels of education are particularly affected by allergies. This is especially the case with middle-aged adults aged between the ages of 30 and 64. The tables present the 12-month prevalence of allergies among 18- to 79-year-old adults stratified by gender, age and educational background (ISCED classification of low, medium and high education [11]).

Allergies are on the one hand caused by genetic factors. On the other hand, various non-genetic factors are discussed to be responsible for the significant rise in allergic disease prevalence that has occurred in recent decades. These factors have long been subject to intensive research and include reduced exposure to microorganisms and infectious agents, reductions in parasitic diseases, increased exposure to allergens, environmental pollution and changes to the intestinal flora, but also changes to diet, lifestyle and travelling patterns [12-14]. The results of the German Health Update show that about one third of adults aged between 18 and 79 years assess themselves as suffering from an allergic condition. The higher prevalence of allergies among women than men and in individuals with the highest levels of education is well-known [15].

Although there was no assessment of single allergic conditions, the results still demonstrate a considerable potential for disease. It can be assumed that each positive response to the study’s question on allergies was also linked to a certain level of psychological strain and (at the very least, symptomatic) medical treatment. However, it has to be noted that laypeople (due to their lack of medical training) often have difficulties in differentiating allergies from ‘pseudo-allergies’ (particularly food intolerances), since both can produce similar symptoms. This is one of the reasons why large-scale national and international epidemiological studies ask questions about specific allergic diseases and often include questions about whether these diseases have been diagnosed by a doctor. Such questions were included in the European ECHRS study (European Community Respiratory Health Survey) and DEGS1 (German Health Interview and Examination Survey for Adults), a representative study of the German adult population, for example. From data of DEGS1, which was conducted between 2008 and 2011 using computer-assisted medical interviews, a 12-month prevalence of nearly 20% for the presence of at least one of seven queried (medically diagnosed) allergies including asthma was estimated [5]. However, estimates of prevalence based on reported medical diagnoses tend to be lower than those ascertained through self-assessments, as many sufferers who have mild symptoms do not visit a doctor.

With respect to time trend in the 12-month prevalence based on self-assessments among adults in Germany, data from three surveys are available for allergic rhinitis (hay fever) [4, 16]. These data demonstrate that the 12-month prevalence almost doubled between 1990/1992 and 2008/2011. Approximately 12.3 million adults in Germany declare to suffer from allergic rhinitis.

Due to the large number of people they affect, allergic diseases are highly relevant to public health. In addition to continuous allergy monitoring and further research on potential risk and protective factors, more efforts are needed to ensure early diagnoses and to develop appropriate forms of care for allergy sufferers. This is not only important for the quality of life of those affected; it is also essential from an economic perspective [8].

## Key statements

- Approximately one third of women but only one quarter of men are currently affected by allergies other than allergic asthma.
- The 12-month prevalence of allergic disease is higher among adults under 65 than among those over 65 years of age.
- Allergic disease is more often reported from adults with the highest levels of education compared to those with lower levels.

## Figures and Tables

**Table 1:** The 12-month prevalence of allergies among 18- to 79-year-old adults, according to gender, age and educational background (n=23,342) Source: GEDA 2014/2015-EHIS

Women	%	(95%-CI)	Men	%	(95%-CI)
**Women total**	**31.6**	**(30.5-32.7)**	**Men total**	**24.5**	**(23.4-25.7)**
**18 – 29 Years**	38.7	(36.4-41.0)	**18 – 29 Years**	31.4	(28.8-34.1)
Low education	36.2	(30.6-42.2)	Low education	29.4	(23.5-36.0)
Medium education	39.8	(36.8-42.9)	Medium education	32.3	(28.7-36.1)
High education	37.9	(33.2-42.9)	High education	32.0	(26.4-38.1)
**30 – 44 Years**	34.8	(32.5-37.1)	**30 – 44 Years**	28.2	(25.9-30.6)
Low education	25.5	(19.6-32.4)	Low education	24.8	(18.2-32.8)
Medium education	35.3	(32.3-38.5)	Medium education	26.4	(23.5-29.5)
High education	38.5	(35.0-42.2)	High education	33.2	(29.9-36.7)
**45 – 64 Years**	32.4	(30.7-34.2)	**45 – 64 Years**	23.6	(22.1-25.3)
Low education	32.6	(28.4-37.0)	Low education	21.6	(17.5-26.4)
Medium education	31.2	(29.0-33.5)	Medium education	22.1	(19.9-24.5)
High education	36.4	(33.6-39.4)	High education	27.3	(24.9-29.9)
**≥ 65 Years**	23.3	(21.2-25.5)	**≥ 65 Years**	16.2	(14.6-18.0)
Low education	21.5	(18.4-24.9)	Low education	15.0	(11.5-19.2)
Medium education	24.8	(21.8-28.1)	Medium education	15.9	(13.5-18.6)
High education	24.1	(20.1-28.7)	High education	17.5	(15.0-20.2)
**Total (women and men)**	**28.1**	**(27.3-29.0)**	**Total (women and men)**	**28.1**	**(27.3-29.0)**

CI=Confidence interval
